# Spontaneous cardiac rupture as the initial presentation of acute myeloid leukaemia complicated by malignant lactic acidosis: a case report

**DOI:** 10.1093/ehjcr/ytaf666

**Published:** 2025-12-22

**Authors:** Yukio Umeda, Yuta Inoue, Shohei Mitta, Yukihiro Matsuno, Kenichiro Azuma

**Affiliations:** Cardiovascular and Thoracic Surgery, Gifu Prefectural General Medical Center, Noishiki 4-6-1, Gifu 500-8717, Japan; Transplantation Research Center, Brigham and Women’s Hospital, Harvard Medical School, 221 Longwood Ave, Boston, MA 02115, USA; Cardiovascular Surgery, Gifu Graduate School of Medicine, Yanagido 1-1, Gifu 501-1194, Japan; Cardiovascular and Thoracic Surgery, Gifu Prefectural General Medical Center, Noishiki 4-6-1, Gifu 500-8717, Japan; Cardiovascular and Thoracic Surgery, Gifu Prefectural General Medical Center, Noishiki 4-6-1, Gifu 500-8717, Japan; Cardiovascular and Thoracic Surgery, Gifu Prefectural General Medical Center, Noishiki 4-6-1, Gifu 500-8717, Japan

**Keywords:** Spontaneous Cardiac Rupture, Left Ventricular, Acute Myeloid Leukemia, Malignant Lactic Acidosis

## Abstract

**Background:**

Left ventricular free wall rupture (LVFWR) without coronary artery occlusion in the setting of acute myeloid leukaemia (AML) is exceedingly rare. We report a rare case of spontaneous cardiac rupture in a patient ultimately diagnosed with acute myeloid leukaemia.

**Case Summary:**

A 77-year-old woman presented with acute chest pain and haemodynamic collapse. Coronary angiography revealed no significant stenosis, whereas left ventriculography demonstrated contrast extravasation from the apical region, consistent with LVFWR. The patient underwent emergent surgery; however, no discrete rupture site was identified. Following temporary weaning from cardiopulmonary bypass and re-inspection, no obvious rupture site was observed. Since it appeared that the blow-out–type rupture had transitioned to an oozing type, suture less repair with TachoSil® was chosen, achieving initial haemodynamic stabilization. Postoperatively, the patient remained haemodynamically stable without persistent bleeding. However, on Postoperative Day 3, the patient was diagnosed with AML, followed by rapidly progressive malignant lactic acidosis and multiorgan failure, leading to death despite intensive care.

**Conclusion:**

This case underscores three key clinical considerations:

(1) In instances of LVFWR without culprit coronary artery occlusion, the possibility of leukaemia-associated myocardial pathology should be actively evaluated.

(2) For oozing-type LVFWR lacking a clearly identifiable rupture site, sutureless repair may represent a feasible and effective surgical option.

(3) The onset or progression of lactic acidosis in the setting of marked leucocytosis constitutes a medical emergency, warranting prompt initiation of cytoreductive therapy and empiric thiamine supplementation.

Learning points

- Consider leukaemia-related myocardial pathology when left ventricular free wall rupture occurs without culprit coronary occlusion.
- Suture less repair may be appropriate when a definite tear is not identifiable and the lesion behaves as oozing-type.
- New or worsening lactic acidosis in leucocytosis should trigger urgent cytoreductive therapy and empiric thiamine, as supportive measures alone are seldom sufficient.

## Introduction

Acute myeloid leukaemia (AML) may involve the heart via leucostasis-related ischaemia or non-ischaemic pathways, notably leukaemic myocardial infiltration that can cause interstitial haemorrhage and structural weakening of the ventricle.^1–3^ Potential mechanisms include leukaemic infiltration with matrix disruption, thrombocytopenia-related intramyocardial haemorrhage evolving into a dissecting haematoma, and inflammatory necrosis from rapid tumour cell lysis; these pathways are supposed to cause spontaneous rupture despite no epicardial coronary occlusion. Yet non–post-infarction left ventricular free wall rupture (LVFWR) in leukaemia is exceptionally uncommon. We present an elderly woman with LVFWR at AML diagnosis, successful sutureless repair, and a postoperative course dominated by malignant lactic acidosis, underscoring leukaemic infiltration as a likely non-ischaemic substrate for rupture.

## Summary figure

**Figure:**
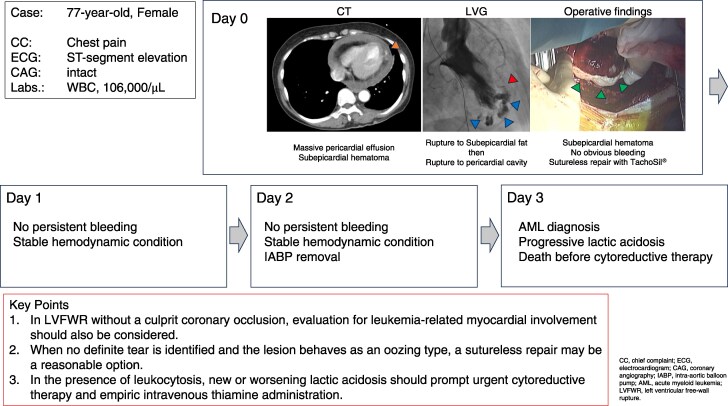


## Case presentation

**Figure 1:**
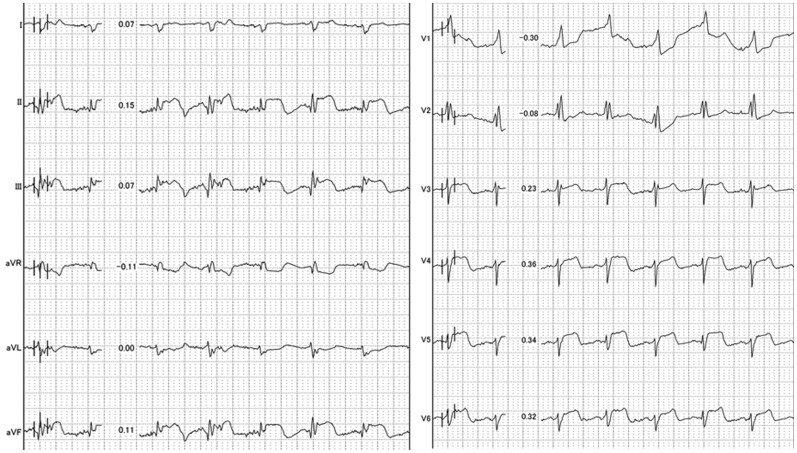
Electrocardiography. On admission, electrocardiography showed ST-segment elevation in leads II, III, and aVF, along with T wave inversion in V1–V3.

**Figure 2:**
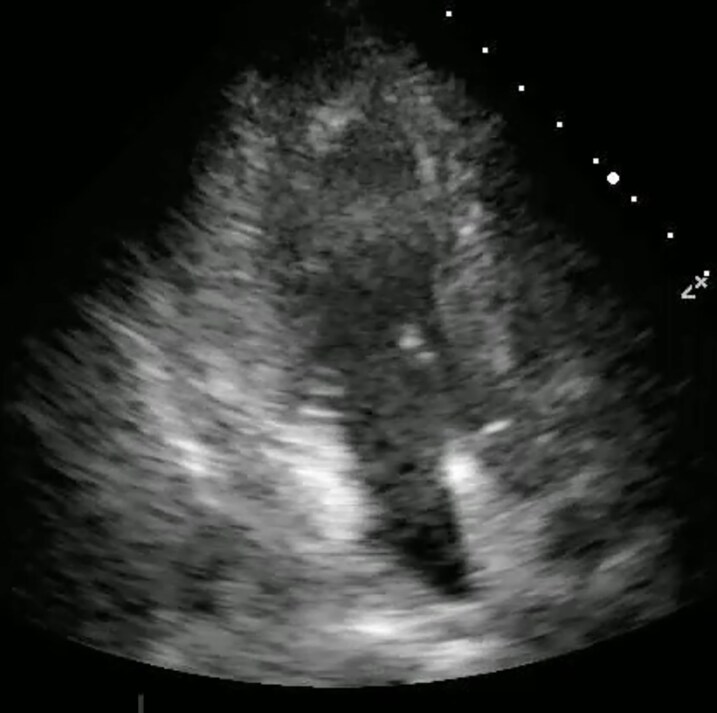
Video_1: transthoracic echocardiography. On admission, transthoracic echocardiography demonstrated moderate pericardial effusion with no distinct regional wall motion abnormalities.

A 77-year-old woman with a history of total gastrectomy for gastric cancer presented to the emergency department (ED) with sudden-onset chest pain. Her vital signs were as follows: blood pressure 71/65 mmHg, heart rate 118 bpm, and oxygen saturation 98% on room air. Electrocardiography showed ST-segment elevation in leads II, III, and aVF and T wave inversion in V1–V3. (*Figure 1*) Transthoracic echocardiography (TTE) revealed moderate pericardial effusion but no apparent regional wall motion abnormalities (*Figure 2*, Supplementary material online, *Video S1*).

**Figure 3:**
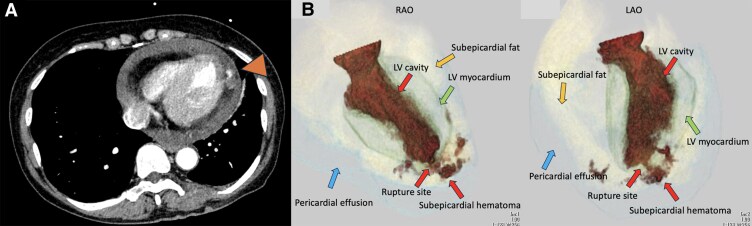
Cardiac CT and cine reconstructions. (*A*) Contrast-enhanced ECG-gated cardiac CT shows a pericardial effusion with contrast extravasation into the subepicardial fat at the left ventricular apex (arrowhead). (*B*) Cine reconstructions in RAO and LAO views delineate the key structures: LV cavity, LV myocardium, subepicardial fat, pericardial effusion, and a subepicardial haematoma/putative rupture site at the apex (colour-coded arrows). CT, computed tomography; RAO, right anterior oblique; LAO, left anterior oblique; LV, left ventricle.

**Figure 4:**
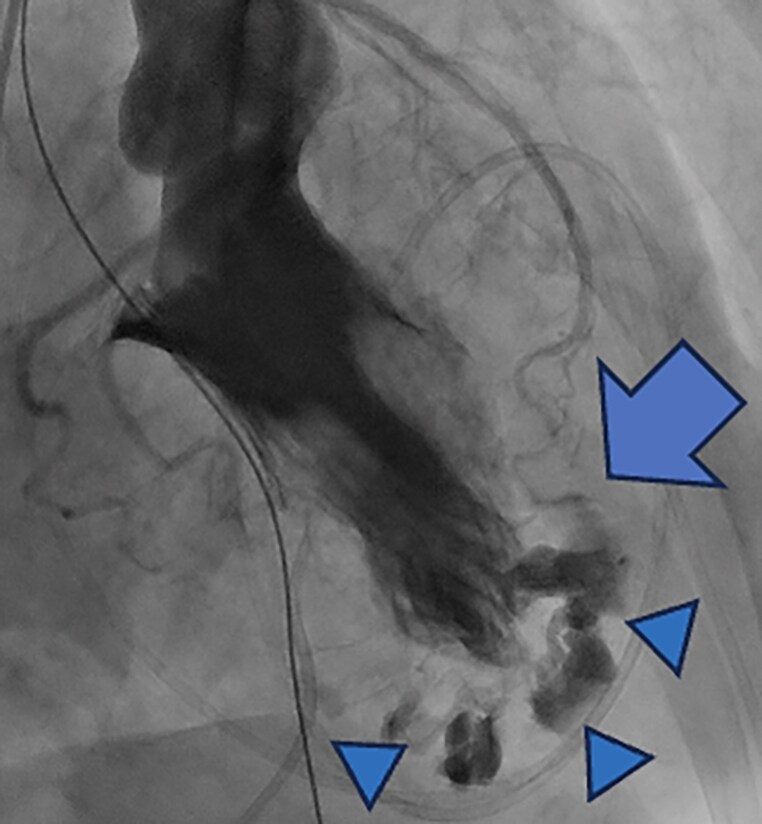
Video 2: left ventriculography. Left ventriculography revealed prominent contrast extravasation into the subepicardial fat tissue at the apex (arrowhead) and further leakage into the pericardial space (arrow), establishing the diagnosis of left ventricular free wall rupture. Video_3: intraoperative findings The patient underwent emergent surgery. After connecting the ECMO cannulas to the cardiopulmonary bypass (CPB) circuit, a median sternotomy was performed. Upon opening the pericardium, a large volume of dark, non-clotted blood was encountered. A supplemental venous cannula was placed via the right atrial appendage. Intraoperative inspection revealed a subepicardial haematoma at the left ventricular apex, but no active bleeding or discrete pericardial rent was identified. After temporary weaning from CPB and repeat inspection, no definite rupture site was seen. These findings suggest that a blow-out–type rupture had evolved to an oozing lesion. Two sheets of TachoSil® were applied to the apical region, and the patient was successfully weaned from CPB with intra-aortic balloon pump support.’ Video_4: postoperative CT findings. Postoperative contrast-enhanced computed tomography (CT) showed no evidence of re-rupture.

Contrast-enhanced computed tomography (CT) demonstrated pericardial effusion and contrast leakage into the subepicardial fat tissue at the apex (*Figure 3*), but no evidence of aortic dissection. Due to haemodynamic collapse, veno-arterial extracorporeal membrane oxygenation (VA-ECMO) was initiated via the right femoral artery and vein. Pericardial drainage was performed, yielding approximately 1500 mL of haemopericardium. Emergent coronary angiography revealed no significant coronary stenosis or occlusion. Left ventriculography (LVG) demonstrated extensive contrast extravasation directly into the subepicardial fat tissue at the apex, with further leakage into the pericardial space (*Figure 4*, Supplementary material online, *Video S2*), confirming the diagnosis of LVFWR. An intra-aortic balloon pump (IABP) was placed via the left femoral artery, and the patient was referred to cardiovascular surgery for emergent repair.

At the time of ED arrival, laboratory tests revealed a markedly elevated white blood cell (WBC) count of 106 000/μL, suggestive of acute leukaemia, with 99% blasts and 1% lymphocytes. Cardiac biomarkers were mildly elevated (CK 55 IU/L, CK-MB 13 IU/L, Troponin T 1.957 ng/mL).

The patient underwent emergent surgery on the same day. After connecting the ECMO cannulas to the cardiopulmonary bypass (CPB) circuit, a median sternotomy was performed. Upon opening the pericardium, a large volume of dark, non-clotted blood was encountered. An additional venous cannula was inserted via the right atrial appendage. Intraoperative inspection revealed a subepicardial haematoma at the left ventricular apex; however, no active bleeding or definite pericardial tear was identified (see Supplementary material online, *Video S3*). Following temporary weaning from CPB and re-inspection, no obvious rupture site was observed. It appeared that the blow-out–type rupture had transitioned to an oozing type. Two sheets of TachoSil® were applied to the apical region, and the patient was successfully weaned from CPB under IABP support.

Postoperatively, the patient remained haemodynamically stable without persistent bleeding. The IABP was removed on Postoperative Day 2 (POD2). However, on POD3, a haematology consult revealed acute myeloid leukaemia based on peripheral smear and immunostaining (MPO-positive blasts). The patient’s condition deteriorated rapidly thereafter, with progressive lactic acidosis and respiratory failure (*Table 1*). CT showed no evidence of re-rupture (Video_4). Despite intensive care including vasopressor support, soon after she was diagnosed as AML, she died on POD3. An autopsy was proposed but was declined.

**Table 1:** Post-operative haemodynamics and acid–base profile

	Day 0			Day 1				Day 2				Day 3		
	10:00	14:00	22:00	4:00	8:00	14:00	22:00	4:00	8:00	14:00	22:00	2:00	8:00	10:35
									IABP removal					Death
Blood gas analysis														
pH	7.532	7.566	7.528	7.492	7.46	7.561	7.476	7.495	7.435	7.491	7.439	7.379	6.932	—
HCO3-, mmol/L	22.3	23.1	24.6	24.3	24.2	22.3	24.3	21.7	23	21.9	18.8	19.4	10.9	—
B.E., mmol/L	0.3	2.1	2.4	1.6	0.8	1.1	1.1	−0.7	−0.8	−0.8	−4.4	−5.1	−20.4	—
Lactate, mmol/L	3.3	3.4	1.9	2.2	2.3	3.4	1.9	2.2	2.3	3.4	4.3	4.9	14	—
pCO2, mmHg	26.7	25.5	29.7	31.5	34.2	24.8	33	28.1	34.3	28.7	27.8	32.9	51.9	—
P/F	262	187	119	285	151	257	185	152	116	157	229	77	98	—
Hemodynamics														
sBP, mmHg	90	80	99	101	110	101	89	87	97	95	97	84	78	—
dBP, mmHg	36	56	46	45	38	45	37	41	47	45	39	35	43	—
HR, bpm	81	60	66	91	65	91	75	75	72	88	84	89	81	—
NTG, μg/kg/min	0.26	0.26	0.26											
CCB, μg/kg/min	1.08				1.08	0.54		1.08	1.89	2.43	1.08			
DOA, μg/kg/min	0.81	0.81	0.81	0.81	0.81	0.81	0.81							
DOB, μg/kg/min											1.62	1.62	8.1	12.15
AD, μg/kg/min														0.27

ABG and lactate values showed rapid progression from mild metabolic acidosis to severe metabolic acidosis with hyperlactataemia on POD3, temporally associated with onset of hypotension and vasopressor initiation. ABG, arterial blood gas; BE, base excess; HR, heart rate; MAP, mean arterial pressure; POD, postoperative day; SpO₂, peripheral oxygen saturation; IABP, intra-aortic balloon pump; POD, postoperative day.

## Discussion

While acute coronary syndrome (ACS) has been occasionally associated with leukaemia due to leucostasis or hypercoagulability,^1,2^ there are also rare reports of spontaneous cardiac rupture without coronary artery occlusion.^3,4^ This phenomenon is analogous to spontaneous splenic rupture seen in patients with leukaemia, particularly AML.^5^ Notably, although only a limited number of clinical cases of cardiac rupture have been documented, early autopsy studies have demonstrated relatively frequent leukaemic infiltration of the myocardium, suggesting that subclinical cardiac involvement may be more common than previously recognized.^6^ Leptidis *et al.* also reported a case assessing fatal cardiac tamponade as the initial manifestation of AML.^7^ Several pathophysiological mechanisms have been proposed to explain spontaneous cardiac rupture in leukaemia: first, AML cells may directly infiltrate the myocardial tissue, disrupting the normal architecture. Second, thrombocytopenia and disseminated intravascular coagulation increase the risk of intramyocardial haemorrhage, which may evolve into a dissecting haematoma or rupture. Finally, rapid lysis of leukaemic cells during induction therapy may result in the release of intracellular contents and pro-inflammatory cytokines, leading to myocardial inflammation, necrosis, or even rupture. In this case, considering CAG did not show culprit coronary occlusion, leukaemic myocardial infiltration with matrix disruption, thrombocytopenia-associated intramyocardial haemorrhage might result in LVFWR.

Surgical repair is the standard of care for LVFWR and should be undertaken without delay.^8^ In this case, unlike typical post-infarction LVFWR, myocardial friability did not correspond to coronary territory, rendering the safety and efficacy of suture repair uncertain. We therefore performed a sutureless repair, treating the lesion as an oozing-type LVFWR. We acknowledge that this management choice is open to debate. However, LVG demonstrated extensive subepicardial contrast extravasation, and the markedly thick subepicardial fat prevented reliable localization of a discrete inflow site, raising substantive concerns about the security of direct suturing.

Lactic acidosis is a medical emergency, which is classified in two types. Type A lactic acidosis is known as tissue depleted of oxygen supply due to hypoperfusion. Type B lactic acidosis occurs without organ hypoperfusion and results of cellular metabolism or a nutritional deficiency, such as thiamine deficiency. In leukaemia, especially AML, it is an oncologic emergency and is frequently refractory unless rapid cytoreduction is achieved.^9^ Proposed mechanisms in AML include Warburg effect,^10^ leukaemic infiltration or dysfunction of the liver or kidneys reduce lactate metabolism,^11^ thiamine deficiency,^12^ and extreme leucocytosis adding a Type A component.^13,14^ In this case, the abrupt rise in lactate on POD3, extreme leucocytosis with 99% blasts, and lack of ongoing bleeding or cardiogenic shock after initial stabilization are most consistent with Type B malignant lactic acidosis due to AML, plausibly compounded by thiamine deficiency (history of total gastrectomy) and evolving microcirculatory impairment from leucostasis. The fulminant course underscores the need for early haematology involvement and rapid cytoreductive measures when malignant lactic acidosis is suspected, even in the immediate postoperative period.

Finally, although the exact pathophysiology remains unknown due to the absence of biopsy or autopsy, this case highlights three clinical messages: (i) consider leukaemia-related myocardial pathology when LVFWR occurs without culprit coronary occlusion; (ii) sutureless repair may be appropriate for oozing-type rupture when a definite tear is not identifiable; and (iii) worsening lactic acidosis in leucocytosis should trigger urgent cytoreductive therapy and empiric thiamine.

## Lead author biography


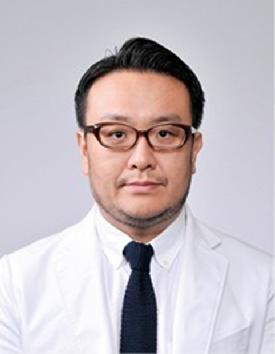


Yukio Umeda, MD, PhD is Chief of Cardiovascular Surgery at Gifu Prefectural General Medical Center, Japan. His clinical specialties include adult cardiac, aortic, and peripheral vascular surgery. He has developed and introduced novel surgical techniques, including the Tailored Stand-up Collar (TSC) technique for acute aortic dissection and the Rafting Anchor Patch (RAP) repair for left ventricular free wall rupture. Dr. Umeda holds board certifications in surgery, cardiovascular surgery, cardiology, angiology, and general thoracic surgery. He also serves as a Councilor of the Japan Association for Thoracic Surgery.

## Supplementary Material

ytaf666_Supplementary_Data

## Data Availability

The data underlying this article are available in the article and in its online Supplementary material.
