# Supplementary material for: Spontaneous cardiac rupture as the initial presentation of acute myeloid leukaemia complicated by malignant lactic acidosis: a case report
Source: Eur Heart J Case Rep. 2025 Dec 22;10(1):ytaf666. doi: 10.1093/ehjcr/ytaf666 (PMC12810419; doi:10.1093/ehjcr/ytaf666)
Supplement: ytaf666_Supplementary_Data [file ytaf666_supplementary_data.zip › [EHJ-CR] Lack of written consent form V1.1 20250821.pdf]

European
Heart Journal
Case Reports

Lack of written consent form

The Editorial Board of *European Heart Journal – Case Reports* believes in the ethical requirement that patients should consent to the publication of their cases. However, it is also appreciated that there are some circumstances where the ability to gain informed consent is not possible or appropriate. For more details about the importance of patient consent for case reports, see Thomson & Camm, Eur Heart J Case Rep 2021 (doi:10.1093/ehjcr/ytaa560).

This form should be used by authors wishing to submit a case report/case series/grand round/images in cardiology article to *European Heart Journal – Case Reports* where written consent is not available for a patient included in the manuscript.

Please use the lack of written consent flow chart to confirm the action that is required. Please provide the information as detailed as possible.

Manuscript Title: Spontaneous Cardiac Rupture as the Initial Presentation of Acute Myeloid Leukemia Complicated by Malignant Lactic Acidosis: a case report

Manuscript ID: EHJ-CR-D-25-01188_R1

Scenario B – The patient is deceased and while there are next-of-kin/surviving relatives, it is not possible to contact them.

B.1. Please outline the reason(s) why the next-of-kin/surviving relative(s) cannot be contacted in this situation (e.g., provide details about which attempts were performed by whom, when, how many times, etc.):

We carefully reviewed “The importance of consent in case reports” and fully understand the importance of obtaining consent.

On November 3, 2025, we verified the contact information recorded in the patient’s medical chart and attempted to contact her relatives by both the registered mobile and home telephone numbers. These calls were made by Dr. Yukio Umeda from our hospital line. However, both telephone numbers were no longer in service.

This case was re-identified during a retrospective review of left ventricular free-wall rupture cases in our department over the past 10 years. It represents an exceedingly rare spontaneous rupture associated with acute myeloid leukemia, recognized during the patient’s treatment course. More than six years have elapsed since this clinical course, and it is presumed that the family has relocated or otherwise changed contact information, rendering the previously recorded numbers inactive.

Given these circumstances, and despite documented attempts to contact the surviving relatives, it has not been possible to obtain consent.

I hereby certify that all information provided in this document is complete, true, and accurate to the best of my knowledge. I acknowledge and accept full responsibility for the correctness of these statements, and I confirm that I have undertaken all reasonable and appropriate measures to comply with the applicable ethical standards, including the obligation to seek and obtain informed patient consent.

Date: November 3, 2025 Signature: Yukio UMEDA

Name of the accountable author: Yukio Umeda
